# Chemical Examination of the Gastric Contents

**Published:** 1905-07-01

**Authors:** 


### CHEMICAL EXAMINATION OF THE GASTRIC CONTENTS.

Dr. W. H. Willcox[1] points out the important relationship of this form of examination to early diagnosis, which, in view of the possibility of surgical interference, has become an increasingly urgent claim. What is wanted is a method having scientific accuracy and capable at the same time of ready clinical application. As the result of experiments Dr. Willcox concludes that one or other of the following is the most suitable "test meal." A pint of weak tea (infused only two or three minutes) with the addition of not more than one oz. of milk, and sugar if desired; a pint of thin arrowroot made with water and about two ozs. of milk, sugar being added to taste; or cornflour, thin gruel or other carbohydrate food may be used instead of arrowroot. With one or other of the above fluids a round of thin buttered toast should be taken; this stimulates the gastric glands and also the pyloric sphincter which consequently prevents the too ready passage of the food into the intestine. The

"test meal" should be given in the early morning before any other food, and should be drawn off after the lapse of one and a half hours. Previous washing out of the stomach is not necessary, but a restricted and light diet should be used for several days in advance. To withdraw the gastric contents after the test meal a long, soft tube should be used with a longer external limb than the part inside the alimentary canal. The syphon action can be started by means of a syringe. The presence of free hydrochloric acid in the stomach contents is of great value in the diagnosis, since the acid is almost invariably absent in carcinoma, whilst it is present in gastric, and also in duodenal ulcer. Those who desire to follow out the actual chemical testing of the stomach contents will find some useful practical directions in Dr. Willcox's paper, but the process of analysis nowadays is usually committed to the clinical laboratory.

[1] Lancet, June 10, 1905.

